# Long-term beta blocker prescribing after myocardial infarction in European primary care (PRACTITIONER)

**DOI:** 10.1186/s12875-026-03208-6

**Published:** 2026-02-09

**Authors:** Martina Zangger, Katharina Tabea Jungo, Limor Adler, Radost Assenova, Olivera Batic-Mujanovic, Luigi Bracchitta, Christine Brütting, Krzysztof Buczkowski, Jelena Danilenko, Patrick Erber, Ileana Gefaell Larrondo, Oksana Ilkov, Katerina Javorska, Aisling A. Jennings, Tonje R. Johannessen, Tuomas Koskela, Donata Kurpas, Vanja Lazić, Stina Mannheimer, Mahmoud Moussa, Martin Seifert, Deona Taraj, Peter Torzsa, Catarina Viegas Dias, Erika Zelko, Baris Gencer, Sven Streit

**Affiliations:** 1 Institute of Primary Health Care (BIHAM), University of Bern, Mittelstrasse 43, Bern, CH-3012 Switzerland; 2 Graduate Health School for Health Sciences, University of Bern, Bern, Switzerland; 3 Center for Healthcare Delivery Science and Division of Pharmacoepidemiology and Pharmacoeconomics, Department of Medicine, Brigham and Women’s Hospital, Harvard Medical School, Boston, MA United States of America; 4 Department of Family Medicine, Gray’s Faculty of Medical & Health Sciences, Tel Aviv University, Tel Aviv, Israel; 5 Department of Urology and General Practice, Faculty of Medicine, Medical University of Plovdiv, Plovdiv, Bulgaria; 6 Department of Family Medicine, Faculty of Medicine, University of Tuzla, Tuzla, Bosnia and Herzegovina; 7 Family Medicine Teaching Center, Public Health Center Tuzla, Tuzla, Bosnia and Herzegovina; 8 Primary Care Department, ATS Città Metropolitana di Milano, Milan, Italy; 9 Institute of General Practice, Faculty of Medicine, University of Halle- Wittenberg, Halle (Saale), Saxony-Anhalt Germany; 10 Department of Family Medicine, Nicolaus Copernicus University in Torun, Torun, Poland; 11 Department of Family Medicine, Rīga Stradiņš University, MFD Health Group, Riga, Latvia; 12 Division General and Family Medicine, Department of General Health Studies, Karl Landsteiner University of Health Sciences, Krems, Austria; 13 Primary Care Research Unit, Gerencia Asistencial de Atención Primaria, Madrid, Spain; 14 Federica Montseny Healthcare Center, Madrid, Spain; 15 Instituto de Investigación Sanitaria Gregorio Marañón, RICAPPS, Madrid, Spain; 16 Department of Family Medicine and Outpatient Care, Medical Faculty, Uzhhorod National University, Uzhorod, Ukraine; 17 Department of Preventive Medicine, Faculty of Medicine in Hradec Králové, Charles University, Hradec Králové, Czech Republic; 18 Department of General Practice, University College Cork, Cork, Ireland; 19 Department of General Practice, Institute of Health and Society, University of Oslo, Oslo, Norway; 20 Faculty of Medicine and Health Technology, Tampere University, Tampere, Finland; 21 The Wellbeing Services County of Pirkanmaa, Tampere, Finland; 22 Division of Research Methodology, Department of Nursing, Faculty of Nursing and Midwifery, Wroclaw Medical University, Wrocław, Poland; 23 Health Center Zagreb - Centar, Zagreb, Croatia; 24 Institute of Health and Care Sciences, University of Gothenburg Centre for Person-Centred Care (GPCC), Sahlgrenska Academy, Gothenburg University, Gothenburg, Sweden; 25 Department of Primary Care Medicine, Medical University of Vienna, Vienna, Austria; 26 Division of General Practice, 3rd Faculty of Medicine, Charles University, Prague, Czechia Czechia; 27 Department of Nursing, Faculty of Health, University of Vlora “Ismail Qemali,” Vlora, Vlorë, Albania; 28 Department of Family Medicine, Semmelweis University, Budapest, Hungary; 29 Comprehensive Health Research Centre (CHRC), NOVA Medical School, NOVA University, Lisbon, Portugal; 30 Institute for General Medicine, Johannes Kepler Universität Linz, Linz, Austria; 31 Service of Cardiology, Lausanne University Hospital, University of Lausanne, Lausanne, Switzerland

**Keywords:** Beta blocker, Myocardial infarction, Preserved left ventricular function, Deprescribing, Primary care

## Abstract

**Background:**

The long-term use of beta blockers after myocardial infarction in patients with preserved ventricular function is debated. General practitioners (GPs) often decide whether to continue or discontinue long-term medications, yet little is known about how they apply evolving evidence to clinical prescribing decisions.

**Objective:**

To assess whether GPs are willing to deprescribe beta blockers post myocardial infarction with preserved left ventricular function and to identify factors associated with deprescribing decisions.

**Design:**

Cross-sectional online survey using case vignettes, conducted between July 2023 and October 2024 in primary care settings in 24 sites across 20 European countries.

**Participants:**

Practicing GPs recruited through convenience sampling at each site.

**Main measures:**

The primary outcome was whether the GP chose to deprescribe beta blockers in the vignettes. Adjusted risk ratios for the association between GP characteristics and the decision to deprescribe were estimated using Poisson regression with generalized estimating equations and robust standard errors, accounting for clustering at the GP and country level.

**Key results:**

604 GPs participated in the survey (median [IQR] age, 44.0 [35.0-54.8] years; 364 [60.3%] female), 89.2% deprescribed beta blockers in at least one vignette. The likelihood of deprescribing increased with time since myocardial infarction (adjusted risk ratio [RR] = 1.28; 95% CI 1.21–1.36 after 5 years; RR = 1.78; 95% CI 1.66–1.90 after 10 years vs. 3 months) and with side effects (RR = 1.76; 95% CI 1.66–1.88). More years of clinical experience were associated with a lower likelihood of deprescribing (RR = 0.86; 95% CI 0.77–0.95 for most vs. least experienced).

**Conclusions:**

In this cross-national vignette study, most GPs were willing to deprescribe beta blockers after myocardial infarction in patients with preserved left ventricular function, particularly when time had passed and side effects were present. These findings suggest that GPs are open to applying evolving evidence on beta blocker discontinuation in clinical care.

**Supplementary Information:**

The online version contains supplementary material available at 10.1186/s12875-026-03208-6.

## Introduction

There is a debate about the long-term prescription of beta blockers after acute myocardial infarction with preserved left ventricular ejection fraction (LVEF) [1]. Since trials in the 1980s demonstrated reduced morbidity and mortality, beta blockers have been a cornerstone of post-myocardial infarction treatment [2, 3]. These trials, however, were conducted before the widespread adoption of modern reperfusion therapies, which have significantly reduced heart failure after myocardial infarction [4]. This raises the question of whether beta blockers benefit patients today, as continued medication use without clear indication may expose patients to adverse drug events and unnecessary harm [5–7]. 

This question has been explored in observational [8–14] and recent interventional studies examining the long-term benefits of beta blocker treatment after myocardial infarction in patients with preserved LVEF with conflicting results [15–20]. According to the European Society of Cardiology, beta blocker therapy should be considered for all patients with acute coronary syndrome regardless of LVEF (class IIa recommendation, Level of Evidence B), without further comment on the duration of the therapy [21]. 

This uncertainty translates into real-world challenges, requiring individualized treatment decisions, particularly in primary care, as most patients with chronic diseases are managed in primary care [22]. General practitioners (GPs) are responsible for deciding whether to continue or adjust long-term treatments, balancing the potential benefits against the risks. This includes deprescribing, defined as a ‘process of withdrawal of an inappropriate medication (…) with the goal of managing polypharmacy and improving outcomes’ by reducing or stopping treatments that may no longer provide clear benefits [23]. In the case of beta blockers after myocardial infarction, GPs must weigh guideline recommendations, evolving clinical evidence and individual patient preferences to ensure patient safety and personalized care [24]. However, guidelines sometimes promote polypharmacy rather than support deprescribing, making it difficult for GPs to reconcile standardized recommendations with individualized treatment decisions, particularly for older patients [25]. While they value evidence, GPs emphasize the need for practical, patient-centered approaches that account for individual circumstances [26]. 

It remains unclear how the current clinical evidence is translated into practice and whether deprescribing beta blockers is considered a viable option in primary care settings. This study examines GPs’ prescribing patterns of beta blockers post myocardial infarction across 20 European countries and identifies the key factors influencing decisions to discontinue therapy.

## Methods

### Study design and study participants

**Fig. 1:**
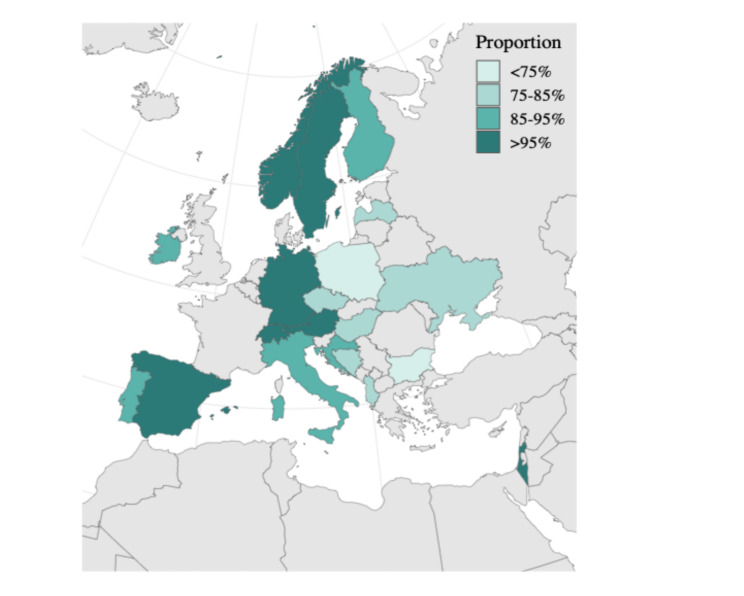
Proportion of general practitioners who decided to deprescribe in at least one of the six case vignettes by country (*n* = 540)

This is a cross-sectional online case vignette survey, conducted among GPs from 20 European countries (see Fig. 1). This study was conducted and reported in accordance with the Checklist for Reporting Results of Internet E-Surveys CHERRIES (see eTable 1). [27]

A national coordinator managed recruitment efforts for each participating site and invited GPs to participate in the survey via email through professional networks, mailing lists, and institutional contacts. They used convenience and snowball sampling, and an open survey design, meaning that any GP with access to the survey link could participate. The national coordinators were recruited through the European General Practice Research Network (EGPRN) and professional contacts of the research team.

### Questionnaire

The survey was developed by the study team at the University of Bern. The electronic questionnaire was piloted with seven clinicians, including six GPs from both academic and non-academic settings and one cardiologist. The national coordinators translated and culturally adapted the questionnaire as needed, with back-translation used to ensure consistency across countries.

The questionnaire consisted of three sections. The first section collected information on GP characteristics, including socio-demographics and work practices. The second section presented three clinical case vignettes describing situations in which a female patient was prescribed a beta blocker after myocardial infarction but had normal LVEF. The first and second vignette differed only in the time since myocardial infarction (three months vs. five years), in the third vignette (10 years after myocardial infarction), the patient also suffered from comorbidities (frailty, Alzheimer’s disease) as presented in eAppendix 1 and eTable 2. All vignettes were first presented without, then with a side effect (dizziness upon standing up). GPs were asked whether they would stop or reduce the beta blocker in each situation, and if they chose to stop or reduce, they were asked which approach they preferred. The third section examined factors influencing beta blocker discontinuation. Participants who reported being willing to deprescribe in the final vignette (10 years after myocardial infarction with side effects present) were asked to select reasons for their decision. Finally, all participants rated the importance of potential factors enabling deprescribing decisions on a 5-point Likert scale.

The questionnaire is presented in eAppendix 2. For the German-speaking questionnaires (Austria, Germany, Switzerland), participants were randomized to view either male or female case vignettes (with the rest of the vignette remaining unchanged). However, the analysis in this paper focuses exclusively on the female case vignettes, as all other versions of the questionnaire included only female case scenarios.

We administered the anonymous survey using SurveyMonkey [28]. Across all sites, the survey was open from July 2023 to October 2024. Participation was voluntary with no incentives, and participants could exit the survey at any time. We used adaptive questioning; deprescribing approach questions appeared only if GPs chose to deprescribe. The survey consisted of 10 to 16 web pages, each displaying between one and ten items. Demographic and vignette questions were mandatory, to ensure completeness. A review step allowed participants to revise their answers. We turned off SurveyMonkey’s ‘Multiple Responses’ option and used cookies to prevent duplicate entries. The ‘Anonymous Responses’ setting ensured that IP addresses were not recorded.

### Statistical analyses

We included all available responses in the analyses. Participants were not required to complete the entire questionnaire to be included; therefore, for each analysis, the denominator reflects the number of participants who answered the respective item (eAppendix 3). We descriptively assessed participants’ sociodemographic characteristics and calculated the crude proportion of GPs who decided to stop or reduce the beta blocker in any of the presented case vignettes to assess the general willingness to deprescribe. For each vignette, we also calculated the crude proportion of GPs who opted to either reduce the dosage or fully stop the beta blocker therapy.

For the main analyses, we used generalized estimating equations with a Poisson distribution with a log link function and robust standard errors, adjusted for the clustering effect at the GP and country level, to estimate relative risks (RR) of the association between the decision to deprescribe and GP characteristics. Due to small sample sizes in most countries, we did not plan to report country-specific results. Lastly, we descriptively analyzed factors influencing deprescribing in the final vignette, reporting proportions for each factor. For Likert-scale responses on deprescribing reasons after myocardial infarction in general, we calculated the crude proportion of responses in each category. We used R (version 4.3.1) to conduct all analyses [29]. 

### Ethics approval and consent to participate

Ethical considerations were addressed according to local requirements in each participating country and in compliance with the Declaration of Helsinki. Most countries did not require ethical approval due to the use of anonymous data, hypothetical cases, and the absence of sensitive information. In Switzerland, we obtained a waiver from the cantonal ethics committee (Req-2022-01057). Where required, the national coordinators obtained it from the respective ethics committees (eTable 3). At the beginning of the survey, we informed participants about its purpose, duration, responsible investigators, and anonymous data collection. Informed consent was implied by participants’ decision to complete the survey.

## Results

A total of 604 GPs from 24 research sites across 20 countries participated in the survey. The median number of responses per country was 25 (IQR 21–34; eTable 4). Among participants, 86.4% (*n* = 522) completed the case vignettes, and 80.1% (*n* = 484) completed the full questionnaire. Table 1 presents the sociodemographic characteristics of the participating GPs. The median age of the 604 participants was 44 years (IQR 35–54), and 60.3% (*n* = 364) were female. The median duration of clinical experience was 12 years (IQR 6–22). Beta blockers after myocardial infarction were most frequently reported to be initiated in a hospital setting (*n* = 521, 90.6%), followed by cardiologists (*n* = 271, 47.1%), GPs themselves (*n* = 105, 18.3%), and others (*n* = 12, 2.1%). Regarding continuation, most GPs perceived themselves as primarily responsible (*n* = 466, 81.0%), followed by cardiologists (*n* = 328, 57.0%), hospitals (*n* = 79, 13.7%), and others (*n* = 11, 1.8%) (total *n* = 575; multiple responses possible).

**Table 1:** Characteristics of participating general practitioners

	Total *n* = 604
Age (years), median (IQR)	44 (35–54)*
Gender, No. (%)	
Female	364 (60.3%)
Male	236 (39.1%)
Non-binary	4 (0.7%)
Experience (years), median (IQR)	12 (6–22) †
Average consultations per full working day, No. (%)	
<15	94 (15.6%)
15–25	246 (40.7%)
26–35	129 (21.4%)
>35	135 (22.4%)
Location of practice, No. (%)	
Urban	385 (63.7%)
Suburban	101 (16.7%)
Rural	118 (19.5%)
Has other medical specialty in addition to GP, No. (%)	131 (21.7%)†
Number of GPs working at practice, No. (%)	
1	147 (24.3%)
≥2	457 (75.7%)
Frequency of discussing patients with colleagues, No. (%)	
Never	14 (2.3%)‡
Rarely (a few times a year)	154 (25.5%)‡
Monthly	124 (20.5%)‡
Weekly	175 (29.0%)‡
Daily	108 (17.9%)‡
Number of GPs who considered deprescribing a beta blocker in the month prior to the survey participation, No. (%)	309 (51.2%)§
Number of GPs who are aware of guidelines regarding the discontinuation of beta blockers, No. (%)	56 (9.3%)‖
GPs’ self-estimated prevalence of …	
Female patients in GPs’ own patient cohort, median (IQR)	56% (51–61%)¶
Patients > 65 years in their own patient cohort, median (IQR)	41% (30–60%)¶
Patients with a beta blocker in their own patient cohort, median (IQR)	20% (10–31%)¶

*Abbreviation*: *GP* General practitioner, *IQR* Interquartile range

* 2 missing

† 1 missing

‡ 7 missing

§ 10 missing

‖ 14 missing

¶ 29 missing

**Fig. 2:**
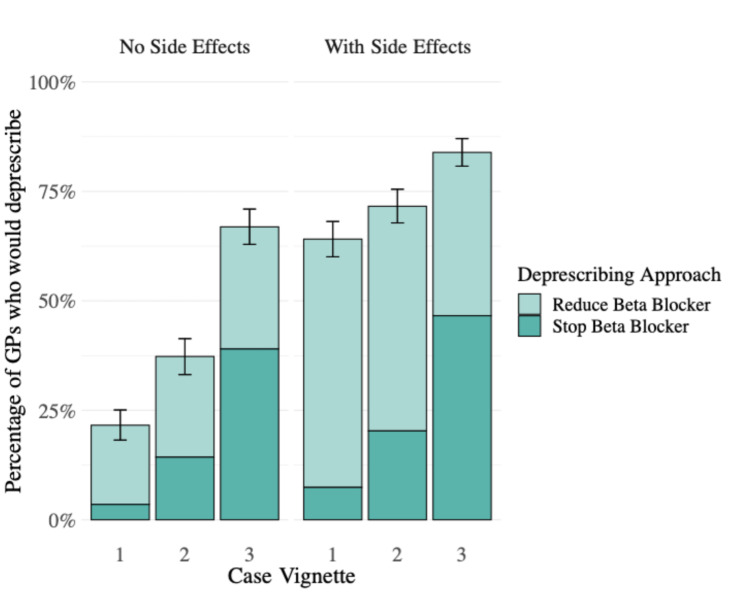
Crude percentages of general practitioners who would deprescribe and the approach they would choose stratified per case vignette and presence of side effects. Vignette 1: Three months after myocardial infarcion; Vignette 2: Five years after myocardial infarctoin; Vignette 3: Ten years after myocardial infarction. GP = general practitioner. Error bars indicate the 95% confidence intervals for the overall deprescribing proportion. Number of replies ranged from 550 in vignette 1 without side effects (no. of missing = 54, 8.9%) to 520 in vignette 3 with side effects (no. of missing = 85, 14.1%)

Overall, 89.2% (*n* = 482 out of 540 who answered at least one vignette scenario) opted to deprescribe in at least one of the six scenarios. Figure 1 displays the proportion of GPs who decided to deprescribe in at least one vignette by country and reveals some differences: the lowest was in Poland (71.1%) and the highest in Austria, Israel, Norway, Spain, Sweden and Switzerland (100%). GPs’ willingness to deprescribe beta blockers varied across the case vignettes, as described in Fig. 2. In the descriptive analysis of the individual vignettes, 21.6% (*n* = 119/550) of GPs opted to deprescribe in Vignette 1 (three months after myocardial infarction) when no side effects were present. Among these, 83.8% (*n* = 98/117) chose to reduce the dosage, and 16.2% (*n* = 19/117) decided to stop the beta blocker entirely. When side effects were present, the percentage of GPs opting to deprescribe increased to 64.1% (*n* = 348/543), with 88.4% (*n* = 306/346) reducing the dosage and 11.6% (*n* = 40/346) stopping the medication. In Vignette 2 (five years after myocardial infarction), 37.3% (*n* = 199/534) of GPs deprescribed without side effects (61.6% (*n* = 122/198) reduced, 38.4% (*n* = 76/198) stopped). This increased to 71.6% (*n* = 379/529) in the presence of side effects (71.6% (*n* = 270/377) reduced, 28.4% (*n* = 107/377) stopped). For Vignette 3 (ten years after myocardial infarction, patient with comorbidities), 66.9% (*n* = 350/532) of GPs deprescribed without side effects (41.7% (*n* = 146/350) reduced, 58.3% (*n* = 204/350) stopped). When side effects were present, 83.9% (*n* = 438/522) deprescribed (44.5% (*n* = 193/434) reduced, 55.5% (*n* = 241/434) stopped).

The regression analysis included 509 GPs with complete data on vignette responses and covariates. GPs were more likely to deprescribe beta blockers as more time elapsed since the myocardial infarction (Table 2). Compared to three months after myocardial infarction, the adjusted risk ratio (RR) for deprescribing was 1.28 (95% CI 1.21–1.36) at five years and 1.78 (95% CI 1.66–1.90) at ten years. Side effects were also significantly associated with an increased likelihood of deprescribing, with an adjusted RR of 1.76 (95% CI 1.66–1.88). In contrast, more clinical experience was associated with a lower likelihood of deprescribing, with an adjusted RR of 0.86 (95% CI 0.77–0.95) for the most experienced GPs compared to the least experienced. Other factors, including gender, number of consultations per day, frequency of professional discussions, practice location and specialization, were not significantly associated with deprescribing.

**Table 2:** Crude and adjusted risk ratios for gps’ willingness to deprescribe beta blockers, adjusted for clustering at the GP and country level (*n* = 509)

Variable	Crude Risk Ratio (95% CI)	*p*-Value	Adjusted Risk Ratio (95% CI)	*p*-Value
Time since myocardial infarction (reference: 3 months after myocardial infarction)				
5 years since myocardial infarction	1.28 (1.21–1.36)	< 0.001	1.28 (1.21–1.36)	< 0.001
10 years since myocardial infarction	1.78 (1.66–1.91)	< 0.001	1.78 (1.66–1.90)	< 0.001
Presence of side effects (reference: no side effects)	1.77 (1.66–1.88)	< 0.001	1.76 (1.66–1.88)	< 0.001
Work experience (reference: low – quartile 1)				
moderate (quartile 2)	0.99 (0.88–1.12)	0.93	0.91 (0.83–1.01)	0.07
high (quartile 3)	0.94 (0.83–1.06)	0.32	0.86 (0.77–0.95)	< 0.001
highest (quartile 4)	0.91 (0.80–1.04)	0.17	0.86 (0.77–0.96)	0.01
Gender = male (reference: female)	1.02 (0.93–1.12)	0.69	0.97 (0.89–1.05)	0.44
≥ 26 Consultations per day (reference < 26)	1.03 (0.94–1.13)	0.50	1.09 (0.99–1.19)	0.07
Frequency of discussing patients with other GPs (reference = less than monthly)				
Monthly	1.14 (0.99–1.30)	0.06	1.03 (0.92–1.16)	0.60
More than monthly	1.09 (0.97–1.23)	0.13	1.04 (0.94–1.16)	0.42
Location of practice (reference = rural)				
suburban	1.14 (1.02–1.28)	0.02	1.02 (0.92–1.15)	0.68
urban	1.08 (0.96–1.21)	0.23	0.99 (0.90–1.09)	0.98
Other Specialization than GP = yes (reference: no)	0.81 (0.70–0.93)	0.002	0. 93 (0.82–1.05)	0.25

*Abbreviations*: *GP* General practitioner. Risk ratios >1 indicate a higher likelihood of deprescribing, while RR <1 indicate a lower likelihood of deprescribing. The model was adjusted for time since myocardial infarction, presence of side effects, work experience (quartiles), GP gender, number of consultations per day, frequency of discussing patients with other GPs, location of practice (rural, suburban, urban), and other specialization than GP

**Fig. 3:**
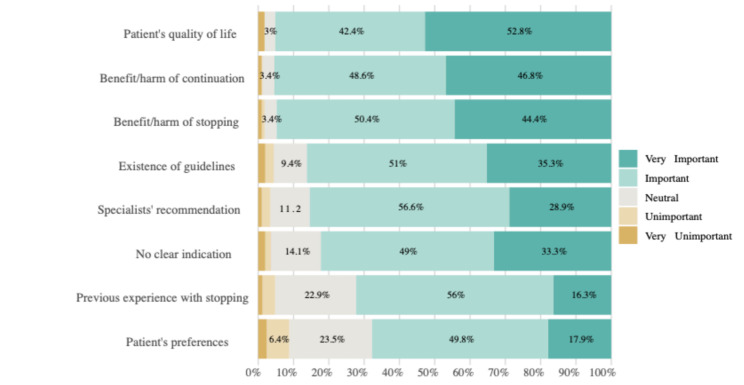
Importance of factors influencing the decision to deprescribe beta blockers (*n* = 502)

The 438 GPs who opted to deprescribe the beta blocker in Vignette 3 (ten years after myocardial infarction with side effects) were asked to specify the reasons for their decision (multiple answers were possible). The most frequently cited reasons were the presence of side effects (88.6%, *n* = 388), followed by frailty (74.4%, *n* = 326) and limited life expectancy (38.6%, *n* = 169). Other reasons included Alzheimer’s disease (32.6%, *n* = 143), patient age (32.2%, *n* = 141), and a lack of indication for continued beta blocker use (26.3%, *n* = 115). All GPs were asked which factors influenced their decision to deprescribe beta blockers after myocardial infarction in general, responses from 502 GPs were available for analysis (Fig. 3). The most important factor was the patient’s quality of life (rated as “very important” by 52.8% (*n* = 265) and “important” by 42.4% (*n* = 213) of respondents). Similarly, the benefit or harm of continuing beta blockers was rated “very important” by 46.8% (*n* = 235) and “important” by 48.6% (*n* = 244), and the benefit or harm of stopping by 44.4% (*n* = 223) and 50.4% (*n* = 253), respectively. Guidelines played a notable role, with 35.3% (*n* = 177) considering them “very important” and 51% (*n* = 256) “important,” while lack of clear indication for continued use was also frequently cited (rated “very important” by 33.3% (*n* = 167) and “important” by 49% (*n* = 246)). In contrast, factors such as specialists’ recommendations, previous experience with deprescribing, and patients’ preferences were rated “very important” by a smaller proportion of respondents (28.9% (*n* = 145), 16.3% (*n* = 82), and 17.9% (*n* = 90), respectively), although many still considered them “important” (56.6% (*n* = 284), 56.0% (*n* = 281), and 49.8% (*n* = 250), respectively).

## Discussion

This study examined whether GPs from 20 European countries were willing to deprescribe beta blockers after myocardial infarction with preserved LVEF and which factors influenced their decisions. Most GPs chose to deprescribe beta blockers in at least one hypothetical vignette. The likelihood of deprescribing increased with time since myocardial infarction and when the patient experienced side effects. The decision to deprescribe was primarily guided by clinical considerations, such as the benefit or harm of stopping beta blockers, and patients’ quality of life.

Beta blocker continuation rates after myocardial infarction in real-world practice show a similar pattern to our findings. Observational studies show that 73% of UK patients continued beta blockers one year after myocardial infarction, but only 50% after three years [30]. In Switzerland, 86% remained on therapy at one year [31], while 72% in Norway did so after three months [32]. An Icelandic study reported 52% of participants remained on beta blockers for secondary prevention of chronic heart disease, though the time since the cardiac event was unspecified [33]. These findings suggest that deprescribing often occurs gradually, potentially in response to evolving patient conditions or clinical reassessments. This aligns with our finding that time since myocardial infarction and the presence of side effects were the most significant factors influencing GPs’ deprescribing decisions. A Swiss observational study further described that after myocardial infarction, older patients with comorbidities were more likely to discontinue secondary prevention medications over time, with adverse effects as the main reason for modifying or discontinuing medications in 63% of cases [31]. Similarly, a cross-sectional survey investigating physicians’ perspectives on deprescribing cardiovascular medications for older adults found that over 90% of physicians deprescribed cardiovascular medications in hypothetical cases when the patient experienced side effects [34]. In contrast, a retrospective UK cohort study that explored the prescribing of beta blockers to patients with cardiovascular disease observed that 70% of patients with adverse effects continued therapy at the same dose [30]. This was believed to be due to already low baseline dosing. Conversely, in our vignettes, there was still room for dose reduction.

Our findings can be further contextualized by recent evidence published after completion of this survey. A recently published meta-analysis pooling five randomized trials found no reduction in mortality, recurrent myocardial infarction, or heart failure when stopping long-term beta blocker therapy in patients with preserved LVEF after myocardial infarction, suggesting that beta blocker discontinuation in this population may be safe [35]. In this context, the willingness of GPs in our study to deprescribe beta blockers appears consistent with this newer evidence, while current clinical guidelines still provide limited guidance on treatment duration or deprescribing.

Finally, we observed that more experienced GPs were slightly less likely to deprescribe beta blockers. This finding contrasts with previous literature suggesting that more experienced GPs tend to prioritize individual patient needs over strict guideline adherence [36]. Similarly, the LESS study, a Europe-wide case vignette study on deprescribing decisions in cardiovascular disease, found that experienced GPs were more willing to deprescribe medications than their younger counterparts [37]. A key difference between our study and LESS is that we focused specifically on beta blockers, whereas LESS examined deprescribing decisions across a broader range of medications in the presence of cardiovascular disease. One possible explanation for our finding is that GPs who trained in earlier decades - when beta blockers were a cornerstone of cardiovascular treatment - may still be influenced by these prescribing patterns, making them more hesitant to deprescribe beta blockers. However, further research would be necessary to confirm this.

Our finding that GPs prioritized patient-centered factors, such as quality of life and the clinical balance of risks and benefits, when making deprescribing decisions aligns with previous research. A Dutch focus group study on GPs’ medication management strategies for polypharmacy patients identified age, prognosis, quality of life, and life expectancy as key considerations in prescribing decisions - factors that also ranked highly in our study [38]. Similarly, a qualitative study in English general practice highlighted that GPs considered a patient’s medical history and comorbidities as central to deprescribing antihypertensive medications. GPs were more confident about withdrawing these medications if patients reported side effects, but without obvious side effects, the lack of clear guidelines was a major barrier to deprescribing [39]. In contrast, a U.S. survey on physicians’ perspectives on deprescribing cardiovascular medications highlighted different challenges, emphasizing patient reluctance and concerns about interfering with other physicians’ treatment plans as major barriers to deprescribing [34]. Interestingly, we found that specialists’ recommendations and patient preferences ranked among the least influential factors in our study.

### Strengths and limitations

This study has several limitations and strengths. Firstly, case vignettes depict hypothetical scenarios and may not fully reflect real-world decisions [40, 41]. However, case vignettes effectively isolate decision-making aspects [42] and elicit responses similar to standardized patients [40, 43]. Secondly, the convenience sampling strategy may have introduced selection bias, potentially limiting generalizability, and did not allow to calculate a response rate. However, it enabled recruitment of a large GP sample from 20 European countries, offering insights into deprescribing across diverse healthcare contexts. Also, some countries were overrepresented, with a disproportionately high number of respondents and as recruitment occurred primarily through academic networks, participating GPs may have been more academically inclined than the general GP population, which may limit the generalizability of our findings. Future studies should aim for a more balanced distribution to improve comparability. Third, findings should be interpreted in the context of the vignette patient, a 68- to 78-year-old female, with normal LVEF, and initially without comorbidities. We chose this design to reflect a GP’s routine practice, where they manage a patient’s care over time as their health evolves. Also, our analysis was restricted to factors explicitly included in the survey, and other unmeasured influences on deprescribing decisions cannot be excluded. Finally, self-reported deprescribing decisions may have introduced social desirability bias, though the anonymous survey aimed to minimize this effect.

### Implications for practice and research

Our findings highlight that deprescribing beta blockers after myocardial infarction is not a one-size-fits-all decision. GPs must navigate the balance between clinical evidence and patient-specific factors, making individualized decisions in the absence of clear guidance. Current evidence and guidelines primarily focus on single diseases and provide limited guidance on deprescribing in multimorbid patients, creating challenges for GPs navigating long-term care [44, 45]. Decision aids and the inclusion of deprescribing recommendations in guidelines that account for multimorbidity could provide GPs with structured yet flexible support [46]. 

## Conclusion

In hypothetical scenarios, GPs are willing to deprescribe beta blockers after myocardial infarction, factoring in patient-specific elements, such as time since myocardial infarction and the presence of side effects. These findings suggest that while the debate around beta blocker continuation persists, GPs are already implementing discontinuation practice in clinical care.

## Supplementary Information

Supplementary Material 1

## Data Availability

The datasets generated and/or analyzed during the current study are not publicly available but are available from the corresponding author on reasonable request. Access will be granted for scientific research purposes following approval by the core study team. A data transfer agreement outlining privacy and data handling obligations must be signed prior to access.

## Declarations

### Prior presentations

This paper was presented at the European General Practice Research Network (EGPRN) meeting on May 10, 2025 and at the Swiss General Internal Medicine (SGIM) Conference on May 21, 2025.
