# Supplementary material for: Long-term beta blocker prescribing after myocardial infarction in European primary care (PRACTITIONER)
Source: BMC Prim Care. 2026 Feb 9;27:93. doi: 10.1186/s12875-026-03208-6 (PMC12983531; doi:10.1186/s12875-026-03208-6)
Supplement: Supplementary file 1 — Supplementary Material 1 [file 12875_2026_3208_MOESM1_ESM.docx]

# Beta Blocker Prescribing after Myocardial Infarction in European Primary Care (PRACTITIONER)

# Supplemental Material

Authors: Martina Zangger, MD^1,2^, Katharina Tabea Jungo, PhD^1,3^, Limor Adler, MD, MPH^4^, Radost Assenova, MD, PhD, DSc^5^, Olivera Batic-Mujanovic, MD, PhD^6,7^, Luigi Bracchitta, MD^8^, Christine Brütting, PhD^9^, Krzysztof Buczkowski, MD, PhD^10^, Jelena Danilenko, MD^11^, Patrick Erber, MD^12^, Ileana Gefaell Larrondo, MD^13,14, 15^, Oksana Ilkov, MD^16^, Katerina Javorska^17^, Aisling A. Jennings, MD, PhD^18^, Tonje R. Johannessen, MD, PhD^19^, Tuomas Koskela^20,21^, Donata Kurpas, MD, PhD^22^, Vanja Lazić, MD^23^, Stina Mannheimer, MD^24^, Mahmoud Moussa, MD^25^, Martin Seifert, MD^26^, Deona Taraj^27^, Peter Torzsa, MD, PhD^28^, Catarina Viegas Dias, MD^29^, Erika Zelko, MD, PhD^30^, Baris Gencer, MD, MPH^1,31^, Sven Streit, MD, PhD, MPH^1^

1. Institute of Primary Health Care (BIHAM), University of Bern, Bern, Switzerland
2. Graduate Health School for Health Sciences, University of Bern, Bern, Switzerland
3. Center for Healthcare Delivery Science and Division of Pharmacoepidemiology and Pharmacoeconomics, Department of Medicine, Brigham and Women’s Hospital and Harvard Medical School, Boston, Massachusetts, United States of America
4. Department of Family Medicine, Gray’s Faculty of Medical & Health Sciences, Tel Aviv University, Tel Aviv, Israel
5. Department of Urology and General Practice, Faculty of Medicine, Medical University of Plovdiv, Plovdiv, Bulgaria
6. Department of Family Medicine, Faculty of Medicine, University of Tuzla, Tuzla, Bosnia and Herzegovina
7. Family Medicine Teaching Center, Public Health Center Tuzla, Tuzla, Bosnia and Herzegovina
8. Primary Care Department, ATS Città Metropolitana di Milano, Milan, Italy
9. Institute of General Practice, Faculty of Medicine, University of Halle-Wittenberg, Halle (Saale), Germany
10. Department of Family Medicine, Nicolaus Copernicus University in Torun, Poland
11. Department of Family Medicine, Rīga Stradiņš University; MFD Health Group, Riga, Latvia
12. Division General and Family Medicine, Department of General Health Studies, Karl Landsteiner University of Health Sciences, Krems, Austria
13. Primary Care Research Unit, Gerencia Asistencial de Atención Primaria, Madrid, Spain
14. Federica Montseny Healthcare Center, Madrid, Spain
15. Instituto de Investigación Sanitaria Gregorio Marañón; RICAPPS, Spain
16. Department of Family Medicine and Outpatient Care, Medical Faculty, Uzhhorod National University, Narodna Square, 3, 88000 Uzhhorod, Transcarpathian Region, Ukraine
17. Department of Preventive Medicine, Charles University, Faculty of Medicine in Hradec Králové, Czech Republic
18. Department of General Practice, University College Cork, Cork, Ireland
19. Institute of Health and Society, Department of General Practice, University of Oslo, Oslo, Norway
20. Faculty of Medicine and Health Technology, Tampere University, Finland
21. The Wellbeing Services County of Pirkanmaa, Finland
22. Division of Research Methodology, Department of Nursing, Faculty of Nursing and Midwifery, Wroclaw Medical University, Wrocław, Poland
23. Health Center Zagreb - Centar, Zagreb, Croatia
24. Institute of Health and Care Sciences, University of Gothenburg Centre for Person-Centred Care (GPCC), Sahlgrenska Academy, Gothenburg University, Sweden
25. Department of Primary Care Medicine, Medical University of Vienna, Vienna, Austria
26. Division of General Practice, 3rd Faculty of Medicine, Charles University, Prague, Czechia
27. Department of Nursing, Faculty of Health, University of Vlora “Ismail Qemali,” Vlora, Albania
28. Department of Family Medicine, Semmelweis University, Budapest, Hungary
29. Comprehensive Health Research Centre (CHRC), NOVA Medical School, NOVA University, Lisbon, Portugal
30. Institute for General Medicine, Johannes Kepler Universität Linz, Linz, Austria
31. Service of Cardiology, Lausanne University Hospital, University of Lausanne, Lausanne, Switzerland

**Corresponding author**: Sven Streit, MSc, PhD, MD, Institute of Primary Health Care, University of Bern, Mittelstrasse 43, CH-3012 Bern, Switzerland ([sven.streit@unibe.ch](mailto:sven.streit@unibe.ch)), [+41 31 684 58 64](tel:+41%2031%20684%2058%2064%20(Assistentin))

**Table of content**

## eTable 1: CHERRIES checklist

## eAppendix 1: Case vignettes as presented in the survey

## eTable 2: Overview over the characteristics of the case vignettes as presented in the survey.

## eAppendix 2: Study questionnaire

**eAppendix 3: Summary of missing data**

## eTable 3: Ethics approval requirements by country - overview of approval necessity and corresponding approvals

## eTable 4: Total number of responses per participating country

## eTable 1: CHERRIES checklist

| **Checklist Item** | **Explanation** | **Page Number** |
| --- | --- | --- |
| Describe survey design | Describe target population, sample frame. Is the sample a convenience sample? (In “open” surveys this is most likely.) | 7-8 |
| IRB approval | Mention whether the study has been approved by an IRB. | 10 |
| Informed consent | Describe the informed consent process. Where were the participants told the length of time of the survey, which data were stored and where and for how long, who the investigator was, and the purpose of the study? | 10 |
| Data protection | If any personal information was collected or stored, describe what mechanisms were used to protect unauthorized access. | 10 |
| Development and testing | State how the survey was developed, including whether the usability and technical functionality of the electronic questionnaire had been tested before fielding the questionnaire. | 8 |
| Open survey versus closed survey | An “open survey” is a survey open for each visitor of a site, while a closed survey is only open to a sample which the investigator knows (password-protected survey). | 8 |
| Contact mode | Indicate whether or not the initial contact with the potential participants was made on the Internet. (Investigators may also send out questionnaires by mail and allow for Web-based data entry.) | 8 |
| Advertising the survey | How/where was the survey announced or advertised? Some examples are offline media (newspapers), or online (mailing lists – If yes, which ones?) or banner ads (Where were these banner ads posted and what did they look like?). It is important to know the wording of the announcement as it will heavily influence who chooses to participate. Ideally the survey announcement should be published as an appendix. | 8 |
| Web/E-mail | State the type of e-survey (eg, one posted on a Web site, or one sent out through e-mail). If it is an e-mail survey, were the responses entered manually into a database, or was there an automatic method for capturing responses? | 9 |
| Context | Describe the Web site (for mailing list/newsgroup) in which the survey was posted. What is the Web site about, who is visiting it, what are visitors normally looking for? Discuss to what degree the content of the Web site could pre-select the sample or influence the results. For example, a survey about vaccination on a anti-immunization Web site will have different results from a Web survey conducted on a government Web site | 9 |
| Mandatory/voluntary | Was it a mandatory survey to be filled in by every visitor who wanted to enter the Web site, or was it a voluntary survey? | 9 |
| Incentives | Were any incentives offered (eg, monetary, prizes, or non-monetary incentives such as an offer to provide the survey results)? | 9 |
| Time/Date | In what timeframe were the data collected? | 9 |
| Randomization of items or questionnaires | To prevent biases items can be randomized or alternated. | 9 |
| Adaptive questioning | Use adaptive questioning (certain items, or only conditionally displayed based on responses to other items) to reduce number and complexity of the questions. | 9 |
| Number of Items | What was the number of questionnaire items per page? The number of items is an important factor for the completion rate. | 9 |
| Number of screens (pages) | Over how many pages was the questionnaire distributed? The number of items is an important factor for the completion rate. | 9 |
| Completeness check | It is technically possible to do consistency or completeness checks before the questionnaire is submitted. Was this done, and if “yes”, how (usually JAVAScript)? An alternative is to check for completeness after the questionnaire has been submitted (and highlight mandatory items). If this has been done, it should be reported. All items should provide a non-response option such as “not applicable” or “rather not say”, and selection of one response option should be enforced. | 9 |
| Review step | State whether respondents were able to review and change their answers (eg, through a Back button or a Review step which displays a summary of the responses and asks the respondents if they are correct). | 9 |
| Unique site visitor | If you provide view rates or participation rates, you need to define how you determined a unique visitor. There are different techniques available, based on IP addresses or cookies or both. | 9 |
| View rate (Ratio of unique survey visitors/unique site visitors) | Requires counting unique visitors to the first page of the survey, divided by the number of unique site visitors (not page views!). It is not unusual to have view rates of less than 0.1 % if the survey is voluntary. | NA |
| Participation rate (Ratio of unique visitors who agreed to participate/unique first survey page visitors) | Count the unique number of people who filled in the first survey page (or agreed to participate, for example by checking a checkbox), divided by visitors who visit the first page of the survey (or the informed consents page, if present). This can also be called “recruitment” rate. | 11 |
| Completion rate (Ratio of users who finished the survey/users who agreed to participate) | The number of people submitting the last questionnaire page, divided by the number of people who agreed to participate (or submitted the first survey page). This is only relevant if there is a separate “informed consent” page or if the survey goes over several pages. This is a measure for attrition. Note that “completion” can involve leaving questionnaire items blank. This is not a measure for how completely questionnaires were filled in. (If you need a measure for this, use the word “completeness rate”.) | 11 |
| Cookies used | Indicate whether cookies were used to assign a unique user identifier to each client computer. If so, mention the page on which the cookie was set and read, and how long the cookie was valid. Were duplicate entries avoided by preventing users access to the survey twice; or were duplicate database entries having the same user ID eliminated before analysis? In the latter case, which entries were kept for analysis (eg, the first entry or the most recent)? | 9 |
| IP check | Indicate whether the IP address of the client computer was used to identify potential duplicate entries from the same user. If so, mention the period of time for which no two entries from the same IP address were allowed (eg, 24 hours). Were duplicate entries avoided by preventing users with the same IP address access to the survey twice; or were duplicate database entries having the same IP address within a given period of time eliminated before analysis? If the latter, which entries were kept for analysis (eg, the first entry or the most recent)? | 9 |
| Log file analysis | Indicate whether other techniques to analyze the log file for identification of multiple entries were used. If so, please describe. | NA |
| Registration | In “closed” (non-open) surveys, users need to login first and it is easier to prevent duplicate entries from the same user. Describe how this was done. For example, was the survey never displayed a second time once the user had filled it in, or was the username stored together with the survey results and later eliminated? If the latter, which entries were kept for analysis (eg, the first entry or the most recent)? | NA |
| Handling of incomplete questionnaires | Were only completed questionnaires analyzed? Were questionnaires which terminated early (where, for example, users did not go through all questionnaire pages) also analyzed? | 9 |
| Questionnaires submitted with an atypical timestamp | Some investigators may measure the time people needed to fill in a questionnaire and exclude questionnaires that were submitted too soon. Specify the timeframe that was used as a cut-off point, and describe how this point was determined. | NA |
| Statistical correction | Indicate whether any methods such as weighting of items or propensity scores have been used to adjust for the non-representative sample; if so, please describe the methods. | 9-10 |

NA = not applicable

## eAppendix 1: Case Vignettes as presented in the survey

Vignette 1)

Your Patient, Mrs. A, is a 68-year-old woman and comes to your practice for a follow-up after she had an **acute ST-elevation myocardial infarction** with placement of a **single drug eluting stent three months ago**. Since then, she takes a dual antiplatelet therapy with **aspirin (100mg/d) and ticagrelor (180mg/d)** as well as **atorvastatin (40mg/d) and metoprolol (100mg/d)** regularly. She does not take any other medication. She has recovered well and **does not suffer from residual symptoms** such as chest pain. Her **left ventricular function is normal with no signs of heart failure or arrythmia**. Otherwise, her medical history is unremarkable, she is retired and spends most of her time looking after her grandchildren. She reports feeling well. Her vital parameters (pulse and blood pressure) and the clinical examination are normal to you. The EKG shows no AV-block.

Vignette 2)

The same patient, Mrs. A., comes **five years later** for a routine follow-up. She is now 73 years old and continues to take **aspirin (100mg/d), atorvastatin (40mg/d) and metoprolol (100mg/d)** regularly. Apart from the myocardial infarction five years ago, her medical history remained unremarkable. Her **left ventricular function is still normal with no signs of heart failure or arrythmia**. She is still **feeling well** and does not suffer from any residual symptoms. Her vital parameters (pulse and blood pressure) and the clinical examination are normal to you. The EKG shows no AV-block.

Vignette 3)

Another **five years later**, Mrs. A, now 78 years old, is moved to a **nursing home**. She has been diagnosed with **Alzheimer’s disease** and is **strongly dependent on support** for her activities of daily living such as dressing, personal hygiene and feeding. She only **hardly walks on the rollator** and has **lost 5kg of weight** in the past six months. She still takes her **aspirin (100mg/d), atorvastatin (40mg/d) and metoprolol (100mg/d)** regularly. Her left ventricular function is still normal with **no signs of heart failure or arrythmia**. Her vital parameters (pulse and blood pressure) are normal to you. The EKG shows no AV-block.

## eTable 2: Overview over the characteristics of the case vignettes as presented in the survey.

| **Vignette** | **Vignette 1:**  **3 months post-MI** | **Vignette 2:**  **5 years post-MI** | **Vignette 3:**  **10 years post-MI** |
| --- | --- | --- | --- |
| **Age** | 68 years | 73 years | 78 years |
| **Setting** | GP practice (follow-up) | GP practice (routine follow-up) | Nursing home |
| **Medical History** | STEMI 3 months ago, DES placement | STEMI 5 years ago | STEMI 10 years ago, Alzheimer’s disease, functional decline |
| **Current Medication** | Aspirin (100 mg/d), Ticagrelor (180 mg/d), Atorvastatin (40 mg/d), Metoprolol (100 mg/d) | Aspirin (100 mg/d), Atorvastatin (40 mg/d), Metoprolol (100 mg/d) | Aspirin (100 mg/d), Atorvastatin (40 mg/d), Metoprolol (100 mg/d) |
| **Left Ventricular Function** | Normal, no heart failure or arrhythmia | Normal, no heart failure or arrhythmia | Normal, no heart failure or arrhythmia |
| **Symptoms** | No residual symptoms, feeling well | No residual symptoms, feeling well | Functionally dependent on support, weight loss, can hardly walk using a rollator |
| **Vital Parameters** | Normal BP and pulse | Normal BP and pulse | Normal BP and pulse |
| **ECG Findings** | No AV-block | No AV-block | No AV-block |

## eAppendix 2: Study questionnaire

1. Welcome!

Thank you for your interest in our project!

We are conducting a survey to investigate general practitioner's (de)prescribing decisions with regards to beta

blocker prescriptions after uncomplicated myocardial infarction at the Institute for Primary Health Care at the

University of Bern, Switzerland.

As there is little recent evidence on this topic, the optimal treatment regarding beta blockers is unclear . We

would like to find out which factors influence your decision to prescribe beta blockers or not and how

prescriptions diﬀer between diﬀerent regions in Europe.

Participation is anonymous and all data will be treated confidentially . The survey is divided into three parts and

should not take more than 10 minutes to complete.

If you agree to participate, please press the "next " button.

Thank you for your support,

Sven Streit, Katharina Jungo and Martina Zangger on behalf of the rest of the study team

2. GP characteristics

First, let us start with a few sociodemographic questions:

1. How old are you (in number of years)?

2. What is your gender identity?

- Woman
- Man
- Non-binary person

3. How many years have you been practicing as GP (in number of years)?

4. How many clinical consultations do you have on average per full working day?

- <15
- 15-25
- 26-35
- >35

5. Where is your practice located at?

- urban
- suburban
- rural

6. Do you have another specialisation apart from GP?

- No
- Yes (please specify)

7. How many general practitioners work at your workplace (inlcuding you)?

- One single GP
- Two or more GPs

8. Make a rough estimate: What percentage of your patients is female?

(If you happen to know the absolute number of female patients in your practice divide that by the total number of patients and multiply by 100 to get the percentage)

9. Make a rough estimate: What percentage of your patients is 65 years or older?

(If you happen to know the absolute number of patients >65 years in your practice divide that by the total number of patients and multiply by 100 to get the percentage)

10. Make a rough estimate: what percentage of your patients is prescribed a beta blocker (for any indication)?

(If you happen to know the absolute number of patients taking a beta blocker in your practice divide that by the total number of patients and multiply by 100 to get the percentage)

3. Work practice

In order for us to understand your daily work practice, please answer the following questions

11. Do you regularly discuss patients with colleagues (e.g., with other GPs in your practice or as part of a quality management program)?

- Never
- Rarely (a few times a year)
- On a monthly basis
- On a weekly basis
- On daily basis

12. In the past month, have you considered discontinuing a beta blocker for any reason for any of your patients?

- Yes
- No

13. Are you aware of any guidelines that address the discontinuation of betablockers after myocardial infarction with preserved left ventricular function?

- No
- Yes, which one

14. In your experience, who starts beta blocker prescription after myocardial infarction? (tick all that apply)

- Hospital where the myocardial infarction was treated initially
- Cardiologist
- General Practitioner
- Other (please specify)

15. Who is responsible for the continuation of beta blocker prescription after myocardial infarction in your region? (tick all that apply)

- Hospital where the myocardial infarction was treated initially
- Cardiologist
- General Practitioner
- Other (please specify)

4. Case vignette 1

In the following, three hypothetical case vignettes are presented. Please specify how you would treat the patient if she came to your practice.

Your Patient, Mrs. A, is a 68-year-old woman and comes to your practice for a follow-up after she had an acute

ST -elevation myocardial infarction with placement of a single drug eluting stent three months ago.

Since then, she takes a dual antiplatelet therapy with aspirin (100mg/d) and ticagrelor (180mg/d) as well

as atorvastatin (40mg/d) and metoprolol (100mg/d) regularly . She does not take any other medication. She

has recovered well and does not suﬀer from residual symptoms such as chest pain. Her left ventricular

function is normal with no signs of heart failure or arrythmia. Otherwise, her medical history is

unremarkable, she is retired and spends most of her time looking after her grandchildren. She reports feeling

well. Her vital parameters (pulse and blood pressure) and the clinical examination are normal to you. The EKG

shows no AV-block.

16. Would you stop or reduce the dosages of her metoprolol?

- yes (stop or reduce)
- no (keep the same dosage)

5. Case vignette 1

(same text) Your Patient, Mrs. A, is a 68-year-old woman and comes to your practice for a follow-up after she

had an acute ST -elevation myocardial infarction with placement of a single drug eluting stent three

months ago. Since then, she takes a dual antiplatelet therapy with aspirin (100mg/d) and ticagrelor

(180mg/d) as well as atorvastatin (40mg/d) and metoprolol (100mg/d) regularly . She does not take any

other medication. She has recovered well and does not suﬀer from residual symptoms such as chest pain.

Her left ventricular function is normal with no signs of heart failure or arrythmia. Otherwise, her

medical history is unremarkable, she is retired and spends most of her time looking after her grandchildren.

She reports feeling well. Her vital parameters (pulse and blood pressure) and the clinical examination are

normal to you. The EKG shows no AV-block.

17. Since you replied "yes" to the previous question, how would you change the dosage?

- reduce dose
- stop medication (= 100% dose reduction, no matter if tapering or direct stop)

6. Case vignette 1

(same text) Your Patient, Mrs. A, is a 68-year-old woman and comes to your practice for a follow-up after she

had an acute ST -elevation myocardial infarction with placement of a single drug eluting stent three

months ago. Since then, she takes a dual antiplatelet therapy with aspirin (100mg/d) and ticagrelor

(180mg/d) as well as atorvastatin (40mg/d) and metoprolol (100mg/d) regularly . She does not take any

other medication. She has recovered well and does not suﬀer from residual symptoms such as chest pain.

Her left ventricular function is normal with no signs of heart failure or arrythmia. Otherwise, her

medical history is unremarkable, she is retired and spends most of her time looking after her grandchildren.

She reports feeling well. Her vital parameters (pulse and blood pressure) and the clinical examination are

normal to you. The EKG shows no AV-block.

18. Would you stop or reduce the dosages of her metoprolol if she were complaining of dizziness when standing up?

- yes (stop or reduce)
- no (keep the same dosage)

7. Case vignette 1

(same text) Your Patient, Mrs. A, is a 68-year-old woman and comes to your practice for a follow-up after she

had an acute ST -elevation myocardial infarction with placement of a single drug eluting stent three

months ago. Since then, she takes a dual antiplatelet therapy with aspirin (100mg/d) and ticagrelor

(180mg/d) as well as atorvastatin (40mg/d) and metoprolol (100mg/d) regularly . She does not take any

other medication. She has recovered well and does not suﬀer from residual symptoms such as chest pain.

Her left ventricular function is normal with no signs of heart failure or arrythmia. Otherwise, her

medical history is unremarkable, she is retired and spends most of her time looking after her grandchildren.

She reports feeling well. Her vital parameters (pulse and blood pressure) and the clinical examination are

normal to you. The EKG shows no AV-block.

19. Since you replied "yes" to the previous question, how would you change the dosage if the patient were complaining of dizziness when standing up?

- reduce dose
- stop medication (= 100% dose reduction, no matter if tapering or direct stop)8. Case vignette 2

8. Case vignette 2

The same patient, Mrs. A., comes five years later for a routine follow-up. She is now 73 years old and

continues to take aspirin (100mg/d), atorvastatin (40mg/d) and metoprolol (100mg/d) regularly . Apart

from the myocardial infarction five years ago, her medical history remained unremarkable. Her left

ventricular function is still normal with no signs of heart failure or arrythmia. She is still feeling well

and does not suﬀer from any residual symptoms. Her vital parameters (pulse and blood pressure) and the

clinical examination are normal to you. The EKG shows no AV-block.

20. Would you stop or reduce the dosages of her metoprolol?

- yes (stop or reduce)
- no (keep the same dosage)

9. Case vignette 2

(same text) The same patient, Mrs. A., comes five years later for a routine follow-up. She is now 73 years old

and continues to take aspirin (100mg/d), atorvastatin (40mg/d) and metoprolol (100mg/d) regularly .

Apart from the myocardial infarction five years ago, her medical history remained unremarkable. Her left

ventricular function is still normal with no signs of heart failure or arrythmia. She is still feeling well

and does not suﬀer from any residual symptoms. Her vital parameters (pulse and blood pressure) and the

clinical examination are normal to you. The EKG shows no AV-block.

21. Since you replied "yes" to the previous question, how would you change the dosage?

- reduce dose
- stop medication (= 100% dose reduction, no matter if tapering or direct stop)

10. Case vignette 2

(same text) The same patient, Mrs. A., comes five years later for a routine follow-up. She is now 73 years old

and continues to take aspirin (100mg/d), atorvastatin (40mg/d) and metoprolol (100mg/d) regularly .

Apart from the myocardial infarction five years ago, her medical history remained unremarkable. Her left

ventricular function is still normal with no signs of heart failure or arrythmia. She is still feeling well

and does not suﬀer from any residual symptoms. Her vital parameters (pulse and blood pressure) and the

clinical examination are normal to you. The EKG shows no AV-block.

22. Would you stop or reduce the dosages of her metoprolol if she were complaining of dizziness when standing up?

- yes (stop or reduce)
- no (keep the same dosage)

11. Case vignette 2

(same text) The same patient, Mrs. A., comes five years later for a routine follow-up. She is now 73 years old

and continues to take aspirin (100mg/d), atorvastatin (40mg/d) and metoprolol (100mg/d) regularly .

Apart from the myocardial infarction five years ago, her medical history remained unremarkable. Her left

ventricular function is still normal with no signs of heart failure or arrythmia. She is still feeling well

and does not suﬀer from any residual symptoms. Her vital parameters (pulse and blood pressure) and the

clinical examination are normal to you. The EKG shows no AV-block.

23. Since you replied "yes" to the previous question, how would you change the dosage if the patient were complaining of dizziness when standing up?

- reduce dose
- stop medication (= 100% dose reduction, no matter if tapering or direct stop)

12. Case vignette 3

Another five years later, Mrs. A, now 78 years old, is moved to a nursing home. She has been diagnosed with

Alzheimer’s disease and is strongly dependent on support for her activities of daily living such as dressing,

personal hygiene and feeding. She only hardly walks on the rollator and has lost 5kg of weight in the past

six months. She still takes her aspirin (100mg/d), atorvastatin (40mg/d) and metoprolol (100mg/d)

regularly . Her left ventricular function is still normal with no signs of heart failure or arrythmia. Her vital

parameters (pulse and blood pressure) are normal to you. The EKG shows no AV-block.

24. Would you stop or reduce the dosages of her metoprolol?

- yes (stop or reduce)
- no (keep the same dosage)

13. Case vignette 3

(same text) Another five years later, Mrs. A, now 78 years old, is moved to a nursing home. She has been

diagnosed with Alzheimer’s disease and is strongly dependent on support for her activities of daily living

such as dressing, personal hygiene and feeding. She only hardly walks on the rollator and has lost 5kg of

weight in the past six months. She still takes her aspirin (100mg/d), atorvastatin (40mg/d) and

metoprolol (100mg/d) regularly . Her left ventricular function is still normal with no signs of heart failure

or arrythmia. Her vital parameters (pulse and blood pressure) are normal to you. The EKG shows no AV-block.

25. Since you replied "yes" to the previous question, how would you change the dosage?

- reduce dose
- stop medication (= 100% dose reduction, no matter if tapering or direct stop)

14. Case vignette 3

(same text) Another five years later, Mrs. A, now 78 years old, is moved to a nursing home. She has been

diagnosed with Alzheimer’s disease and is strongly dependent on support for her activities of daily living

such as dressing, personal hygiene and feeding. She only hardly walks on the rollator and has lost 5kg of

weight in the past six months. She still takes her aspirin (100mg/d), atorvastatin (40mg/d) and

metoprolol (100mg/d) regularly . Her left ventricular function is still normal with no signs of heart failure

or arrythmia. Her vital parameters (pulse and blood pressure) are normal to you. The EKG shows no AV-block.

26. Would you stop or reduce the dosages of her metoprolol if she were complaining of dizziness when standing up?

- yes (stop or reduce)
- no (keep the same dosage)

15. Case vignette 3

(same text) Another five years later, Mrs. A, now 78 years old, is moved to a nursing home. She has been

diagnosed with Alzheimer’s disease and is strongly dependent on support for her activities of daily living

such as dressing, personal hygiene and feeding. She only hardly walks on the rollator and has lost 5kg of

weight in the past six months. She still takes her aspirin (100mg/d), atorvastatin (40mg/d) and

metoprolol (100mg/d) regularly . Her left ventricular function is still normal with no signs of heart failure

or arrythmia. Her vital parameters (pulse and blood pressure) are normal to you. The EKG shows no AV-block.

27. Since you replied "yes" to the previous question, how would you change the dosage if the patient were complaining of dizziness when standing up?

- reduce dose
- stop medication (= 100% dose reduction, no matter if tapering or direct stop)

28. What are your reasons for stopping or reducing the metoprolol in this situation? (multiple answers possible)

- her age
- frailty (in her case defined by the unintentional weight loss, low level of activity , slow gait speed)
- Alzheimer’s disease
- limited life expectancy
- lack of indication
- side-eﬀects (in her case dizziness when standing up)
- other (please specify)

16. Relevant factors for discontinuing or reducing beta blockers

Last, we would like to know which factors are important for you, when you are

deciding if you want to stop or reduce a beta blocker29. How important are the following factors when you consider stopping or reducing a beta blocker? (Very unimportant, unimportant, neutral, important, very important)

- Existence of guidelines to stop/reduce
- No clear indication
- Recommendations from involved specialists
- Benefit/harms of continuing
- Benefit/harms of stopping
- Previous experiences when stopping or reducing
- Patient’s preference
- Patient’s quality of life

Are there other factors that are important for you when you consider stopping or reducing a beta blocker?

(please specify)

**eAppendix 3: Summary of missing data**

To assess data completeness, we examined the number and proportion of missing responses across the three main sections of the questionnaire. The total number of participants was 604.

1. Demographic Characteristics

- Overall, 31 of 604 participants (5.1%) had at least one missing value in this section.
- The number and proportion of missing responses for each item were as follows:
  - Age: 2 missing (0.3%)
  - Years of clinical experience: 1 missing (0.2%)
  - Gender: complete
  - Location of practice (urban/suburban/rural): complete
  - Number of GPs at the practice: complete
  - Average number of consultations per day: complete
  - Specialty in addition to GP: 1 missing (0.2%)
  - Frequency of discussing patients with colleagues: 7 missing (1.2%)
  - Considered deprescribing a beta blocker in the month prior: 10 missing (1.7%)
  - Awareness of deprescribing guidelines: 14 missing (2.3%)
  - Self-estimated prevalence of female patients: 29 missing (4.8%)
  - Self-estimated prevalence of patients >65 years: 29 missing (4.8%)
  - Self-estimated prevalence of patients with a beta blocker: 29 missing (4.8%)

2. Case Vignettes

Participants were presented with three clinical case vignettes, each in two versions: one without side effects and one with a side effect (dizziness). For each scenario, GPs were asked whether they would deprescribe beta blockers. If they chose to deprescribe, they were then asked to indicate how they would proceed (dose reduction or complete discontinuation).

- Complete vignette responses (all six scenarios answered were available for 519 out of 604 participants (85.9%)
- A total of 540 out of 604 (89.4%) participants answered at least one of the six vignette scenarios.
- The number of missing responses per vignette is shown below. For each vignette, the number and percentage of missing responses to the deprescribing approach question is also reported (denominator: only those who chose to deprescribe in that vignette).

| **Vignette Scenario** | **Missing vignette response (n, %)** | **Chose to deprescribe (n)** | **Missing approach response (n, %)** |
| --- | --- | --- | --- |
| Vignette 1 – No side effects | 54 (8.9%) | 119 | 2 (1.7%) |
| Vignette 1 – With side effects | 61 (10.1%) | 348 | 2 (0.9%) |
| Vignette 2 – No side effects | 70 (11.6%) | 199 | 1 (0.5%) |
| Vignette 2 – With side effects | 75 (12.4%) | 379 | 2 (0.5%) |
| Vignette 3 – No side effects | 81 (13.4%) | 350 | 0 (0.0%) |
| Vignette 3 – With side effects | 85 (14.1%) | 438 | 4 (0.9%) |

3. Reasons to Deprescribe

In the final part of the questionnaire, all participants were asked to rate how strongly various factors influenced their decision to deprescribe beta blockers after AMI in general.

- Complete responses were available for 502 out of 604 participants (83.1%).
- A total of 102 participants (16.9%) did not respond to this section

## eTable 3: Ethics approval requirements by country - overview of approval necessity and corresponding approvals

| **Country** | **Ethics Approval** |
| --- | --- |
| Albania | Approval obtained (No. 280/2) |
| Austria | Not required |
| Bosnia and Herzegovina | Approval obtained (EK-03-011-CS/25) |
| Bulgaria | Not required |
| Croatia | Not required |
| Czech Republic | Approval obtained from the Ethics Committee of the 3rd Faculty of Medicine, Charles University |
| Finland | Not required |
| Germany | Not required |
| Hungary | Not required |
| Ireland | Approval obtained (2024-110) |
| Israel | Approval obtained (No. 0007027.1) |
| Italy | Not required |
| Latvia | Approval obtained (2-PĒK-4/90/2024) |
| Norway | Not required |
| Poland | Approval obtained (KB 474/2024) |
| Portugal | Approval obtained (1317/CES/2024) |
| Spain | Approval obtained (Noº CEIm: 23/394 ) |
| Sweden | Not required |
| Switzerland | Waiver obtained (2022-01057) |
| Ukraine | Not required |

## eTable 4: Total number of responses per participating country

|  | Total n = 604 |
| --- | --- |
| **Country** | **No. (%)** |
| Albania | 20 (3.3%) |
| Austria | 27 (4.5%) |
| Bosnia and Herzegovina | 37 (6.1%) |
| Bulgaria | 22 (3.6%) |
| Croatia | 24 (4.0%) |
| Czech Republic | 53 (8.8%) |
| Finland | 20 (3.3%) |
| Germany | 33 (5.5%) |
| Hungary | 23 (3.8%) |
| Ireland | 21 (3.5%) |
| Israel | 26 (4.3%) |
| Italy | 28 (4.6%) |
| Latvia | 22 (3.6%) |
| Norway | 52 (8.6%) |
| Poland | 41 (6.8%) |
| Portugal | 62 (10.3%) |
| Spain | 21 (3.5%) |
| Sweden | 20 (3.3%) |
| Switzerland | 31 (5.1%) |
| Ukraine | 21 (3.5%) |
